# Effects of V, Nb, Si, Mn, Mo on Microstructural Evolution and Strength–Toughness Balance of P20 Plastic Mold Steel

**DOI:** 10.3390/ma19173649

**Published:** 2026-08-27

**Authors:** Luliang Zhao, Ziwen Li, Zhenguo Hou, Min Yang, Chunqiao Xing, Jie Yan, Zan Yao

**Affiliations:** 1 School of Materials Science and Engineering, Shanghai University, Shanghai 200444, China; 18115694113@163.com (L.Z.); ziwenli2025@163.com (Z.L.); 13673319742@163.com (Z.H.); 18642515957@163.com (C.X.); 19732612778@163.com (J.Y.); 2 Composite Research Center, Shanghai University, Shanghai 200444, China; ym2008@shu.edu.cn; 3 State Key Laboratory of Advanced Refractories, Shanghai University, Shanghai 200444, China

**Keywords:** plastic mold steel, carbide precipitates, microstructure, mechanical properties, strengthening mechanisms

## Abstract

With the continuous development of plastic products toward larger dimensions, higher precision, and extended service life, plastic mold steels are required to simultaneously possess superior wear resistance, strength, and toughness. The effects of five alloying elements (V, Nb, Si, Mo, and Mn) on the microstructural evolution and mechanical properties of P20 plastic mold steel were systematically investigated after air-cooling from 860 °C, followed by tempering at 525 °C, and the underlying strengthening and toughening mechanisms were elucidated. The results revealed that, in the 0.2 V steel, approximately 62.6% of V existed in the form of fine VC carbides after austenitization at 860 °C, effectively inhibiting austenite grain coarsening. The remaining dissolved V atoms subsequently precipitated as nanoscale V–Mo-rich MC-type carbides during tempering, with an average size of less than 50 nm. This precipitation strengthening contributed an estimated strengthening increment of approximately 760 MPa, corresponding to a measured tensile strength increase of 326 MPa relative to the P20. In contrast, in the 0.1 Nb specimen, solubility calculations indicate that over 99% of Nb remains in undissolved NbC particles; TEM observations show these particles range from coarse 1–3 μm to finer 100–200 nm in size. The contribution of coarse NbC particles to material strength improvement is limited. The addition of Mo promoted the formation of abundant nanoscale MoC-type carbides (2–10 nm), which also exhibit a notable precipitation strengthening effect. Meanwhile, Si mainly contributed to solid-solution strengthening, whereas Mn enhanced the strength through solid-solution strengthening and grain refinement. Charpy impact tests demonstrated that, despite the remarkable strengthening induced by nanoscale carbide precipitation in the 0.2 V steel (tensile strength: 1237 MPa), the impact toughness deteriorated severely, dropping to 21 J. This severe toughness loss is proposed to be associated with local stress concentration around the fine carbides, which promotes secondary crack propagation. Similarly, coarse micrometer-sized NbC particles acted as detrimental sites for crack initiation and impaired impact toughness. Comparative analysis indicated that the steels containing 0.75 wt.% Si, 0.7 wt.% Mo, and 1.5 wt.% Mn achieved a favorable balance between strength, ductility, and toughness. In particular, the 0.7 Mo steel exhibited the most outstanding combination of mechanical properties, attaining a tensile strength of 1207 MPa and an impact energy of 136 J.

## 1. Introduction

Plastic products play an irreplaceable role in modern industry and daily life, with their forming quality and production efficiency highly dependent on the performance of plastic mold steel. P20 plastic mold steel, due to its excellent hardenability, machinability, and reasonable cost advantages, is widely used in the manufacturing of injection molding tools for large household appliances, automotive components, and precision electronic instrument housings [1,2]. As plastic products continue to evolve toward larger size, higher precision, and longer service life, the operating conditions for molds are becoming increasingly severe. This not only demands that plastic mold steels possess sufficient strength and hardness to resist deformation and wear under repeated loading, but also requires excellent ductility and toughness to withstand impact loads induced by complex thermal–mechanical cycles [3,4]. Therefore, how to further improve the strength and hardness of plastic mold steels without greatly sacrificing toughness has become a core issue of long-term concern in mold steel research and development.

Alloying control is one of the most effective methods for improving the comprehensive mechanical properties of plastic mold steels. Vanadium (V) possesses a relatively high solubility in austenite, exhibits pronounced precipitation strengthening effects, and is easily controllable, making it widely recognized as one of the most effective alloying elements. Wang et al. [5] showed that adding 0.13 wt.% V to 20Mn2SiCrS steel can broaden the cooling range for bainitic transformation, enabling the formation of granular bainite after continuous cooling following forging, thereby achieving excellent mechanical properties. Yuan et al. [6] demonstrated that by controlling the V content in 55NiCrMoV7 mold steel, VC particles with high activation energy can precipitate within the matrix, pinning grain boundaries and dislocations, thereby enhancing thermal–mechanical fatigue performance. Nb elements greatly improve the strength and toughness of ultra-high-strength steels through synergistic effects of precipitation strengthening and grain refinement. Mohammed Ali et al. [7] found that adding 0.04 wt.% Nb can stabilize and expand the ferrite phase region, slightly increase the critical transformation temperature (Ac3), refine the effective grain size, and moderately improve strength. However, the presence of coarse precipitates and high-density inclusions deteriorates impact toughness. Jiang et al. [8] showed that the impact toughness of X80 steel is closely related to the Nb content. Steel with 0.05 wt.% Nb has a lower M/A constituent content and a higher proportion of high-angle grain boundaries, whereas steel with 0.08 wt.% Nb exhibits the opposite characteristics. Since impact cracks tend to initiate near the M/A constituents, high-angle grain boundaries can greatly suppress crack propagation, resulting in better impact toughness for the steel containing 0.05 wt.% Nb. In addition to V and Nb, Si, Mo, and Mn serve as fundamental alloying elements in P20 plastic mold steel, and their content control also greatly influences the microstructure and properties [9,10,11,12,13]. While individual alloying effects in plastic mold steels have been studied, a systematic comparative evaluation of specific P20 modifications under a common heat-treatment route has not been reported. Such a comparative assessment is practically valuable for industrial alloy selection and provides insights into the differential strengthening and toughening responses of these modifications under identical processing conditions.

To reveal the differential strengthening mechanisms of various alloying elements and ensure the validity of comparative studies, this research specifically designed the content of the added elements. A small amount of V can greatly improve the strength, toughness, and wear resistance of plastic mold steel [14]. However, excessive V leads to non-uniform distribution of V in the matrix, forming VC eutectic carbides that reduce the impact toughness of the steel [15,16]. Huang et al. [17] showed that as the V content increases, the thermal conductivity and thermal stability of mold steel decrease. A V content of 0.55 wt.% causes carbide coarsening and reduces mechanical properties, while 0.15 wt.% V provides better thermal stability and mechanical performance. Therefore, in this study, 0.2 wt.% V was added as a variable. Most existing studies have focused on the effects of low-Nb elements on alloy properties [18,19,20], while the presence form and impact of high-Nb content in plastic mold steels remain unclear. Therefore, the Nb content in this study was selected as 0.1 wt.%. The Si content was increased from 0.25 wt.% to 0.75 wt.%, achieving pronounced solid solution strengthening while avoiding toughness degradation caused by excessive Si. The Mo content was increased from the base level of 0.35 wt.% to 0.7 wt.% with the aim of evaluating its contribution to nano-scale MoC precipitation strengthening beyond that achieved with the conventional composition. The Mn content was increased from 0.7 wt.% to 1.5 wt.%, achieving high hardenability while avoiding excessive segregation caused by excessively high Mn levels.

This study is based on P20 plastic mold steel as the base composition, with V, Nb, Si, Mo, and Mn elements either added or controlled to investigate the effect of alloying elements on hardness at different tempering temperatures. Under a unified pre-hardening process (austenitizing at 860 °C followed by air cooling and tempering at 525 °C), the effects of different alloying elements on microstructure and mechanical properties were systematically investigated to provide experimental evidence and theoretical guidance for optimizing the composition and microstructure design of high-strength and high-toughness plastic mold steels.

## 2. Materials and Methods

All steel ingots were melted in a ZGJL0.01-50-4K vacuum induction melting furnace (rated capacity: 6 kg; maximum operating temperature: 1600 °C) under a protective argon atmosphere. To eliminate as-cast defects generated during solidification and to reduce microsegregation, the ingots were subjected to high-temperature homogenization and hot forging. The specific procedure was as follows: the ingots were heated in the furnace to 1100 °C and held for 1 h to achieve sufficient compositional homogenization; subsequently, multi-pass hot forging was carried out in the temperature range of 940–1060 °C, and the ingots were finally forged into square billets with a cross-sectional dimension of 30 mm × 30 mm.

The experiment used P20 plastic mold steel as the reference steel, with V, Nb, Si, Mo, and Mn elements added or adjusted accordingly. The specific chemical compositions are shown in Table 1. Heat treatment was performed in a KSL-1500X-S box-type resistance furnace (Hefei Kejing Materials Technology Co., Ltd., Hefei, China) with a temperature uniformity of ±5 °C across the working zone, verified by calibration against a type-K thermocouple prior to each run. Specimens (19 mm × 19 mm × 20 mm) were placed on a ceramic plate with a spacing of ≥20 mm between specimens to ensure uniform thermal exposure. The heating rate was 10 °C/min to the target temperature. The holding time was started when the thermocouple (placed adjacent to the specimens) reached the set temperature. For austenitizing at 860 °C, the holding time was 45 min, followed by air cooling to room temperature on a metal grid with a cooling rate of approximately 15–20 °C/min (measured by thermocouple attached to a dummy specimen). For tempering, specimens were heated at 10 °C/min to 475–550 °C, held for 60 min, and air-cooled.

After grinding and polishing the heat-treated sample, we etched it with a 4% nitric acid alcohol solution for 10–15 s to reveal the microstructure. For prior austenite grain boundary observation, samples were etched with a saturated picric acid–alcohol solution (5 g picric acid in 100 mL ethanol plus 1 mL of sodium dodecylbenzene sulfonate as a wetting agent) at room temperature for 60–120 s, followed by gentle wiping with cotton soaked in ethanol to remove the etchant residue. Microstructural analysis of the different alloyed samples was conducted using a BH200M optical microscope (OM, Ningbo Sunny Instruments Co., Ltd., Ningbo, China), an FEI Nova Nano SEM 450 scanning electron microscope (SEM, FEI Company, Hillsboro, OR, USA) equipped with an energy-dispersive spectrometer (EDS), and a Talos F200X G2 transmission electron microscope (TEM, Thermo Fisher Scientific, Waltham, MA, USA). EBSD was employed to analyze the microstructural and crystallographic differences among the various alloyed samples.

TEM specimens were prepared as thin foils by mechanical grinding to 50 μm followed by twin-jet electropolishing with an electrolyte of 10% perchloric acid in ethanol at −30 °C and a voltage of 30 V. Foils were examined in a Talos F200X G2 transmission electron microscope operated at 200 kV. SAED patterns were acquired using a camera length of 250 mm, calibrated against a standard Au polycrystalline specimen. EDS analysis was performed in STEM mode with a Super-X EDS detector system. Undissolved particles after austenitization were distinguished from secondary precipitates formed during tempering by comparing as-austenitized/air-cooled specimens (without tempering) and tempered specimens (525 °C). Particles present in the as-austenitized/air-cooled condition that persisted through the tempering cycle were classified as undissolved particles, while particles newly observed only in the tempered condition were classified as tempering-induced precipitates.

EBSD analysis was performed on an FEI Nova Nano SEM 450 equipped with EBSD detector. Specimens for EBSD were mechanically polished to 1 μm diamond finish followed by vibratory polishing with colloidal silica (0.04 μm) for 4 h to remove surface deformation. EBSD data were acquired at an accelerating voltage of 20 kV, working distance of 15 mm, sample tilt of 70°, and step size of 0.3 μm. The mapped area was 80 μm × 80 μm per field, with 1 field per specimen and 3 specimens per condition. The indexing rate was typically >92%. Data were processed using AZtecCrystal software (Version 2.1). Grain boundaries were defined using a misorientation threshold of 15° for high-angle grain boundaries and 2–15° for low-angle grain boundaries. The minimum grain size was set to 3 pixels. KAM maps were calculated using a kernel of the first-neighbor order with a maximum misorientation cutoff of 5°.

Tensile tests were conducted at room temperature using a universal tensile testing machine, with a gauge length of 25 mm and a diameter of 5 mm, at a crosshead speed of 0.5 mm/min. Charpy impact tests with V-notch were performed at room temperature using a pendulum impact tester, with specimen dimensions of 10 mm × 10 mm × 55 mm. For each group of specimens in the tensile and impact tests, three repeated trials were carried out. Rockwell hardness was measured for different alloyed specimens using a Rockwell hardness tester, with ten repeated measurements per specimen group and the average value taken. Mechanical properties were evaluated according to the following standards: Rockwell hardness (HRC): GB/T 230.1-2018 [21]. Tensile testing: GB/T 228.1-2021 [22]. Charpy V-notch impact testing: GB/T 229-2020 [23]. For longitudinal cross-sectional observation of impact fracture surfaces, specimens were sectioned perpendicular to the fracture surface along the central plane of the Charpy specimen, passing through the notch root. The cutting plane was located at the mid-thickness of the specimen. As with the heat-treated specimen, this was embedded and etched with 4% nitric acid alcohol solution.

Effective grain size in this study refers to the mean equivalent diameter of martensite/bainite blocks (regions separated by boundaries with misorientation > 15°), as measured by EBSD. This threshold was chosen based on prior work showing that boundaries with misorientation > 15° effectively block cleavage crack propagation and thus represent the effective microstructural unit for toughness. The equivalent diameter was calculated as the diameter of a circle having the same area as the measured block. ‘Prior austenite grain size’ (measured by optical microscopy) and ‘effective grain size’ (measured by EBSD) are distinct quantities and are not used interchangeably.

## 3. Results and Discussion

### 3.1. Tempering Hardness of Different Steels

**Figure 1:**
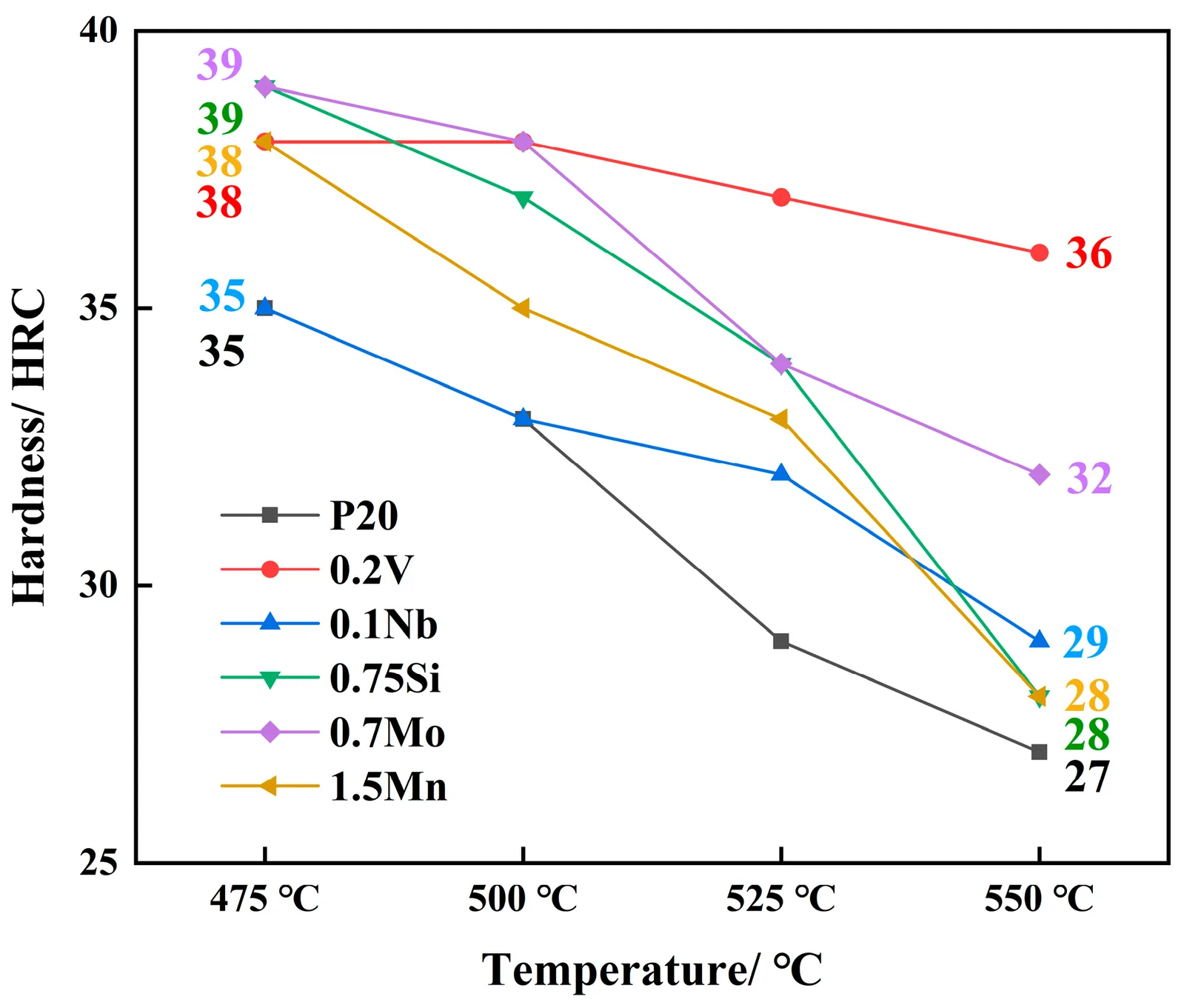
Tempering hardness distribution curves of different alloyed specimens.

Figure 1 shows the hardness distribution curves of six different alloyed specimens at various tempering temperatures. As the tempering temperature increases, the hardness of all specimens decreases, but there are differences in the effect of various alloying elements on tempering softening resistance. Within the tempering temperature range of 475 °C to 550 °C, the samples with 0.2 V, 0.1 Nb, and 0.7 Mo exhibited strong tempering stability, with hardness reductions of 2 HRC, 6 HRC, and 7 HRC, respectively. The hardness of the 0.2 V sample remained almost constant, indicating that the addition of V greatly enhanced the matrix’s resistance to temper softening. In contrast, the hardness of the samples with 0.75 Si, 1.5 Mn, and the original P20 composition decreased more rapidly, with reductions reaching 11 HRC, 10 HRC, and 8 HRC, respectively, suggesting that Si and Mn have limited effects on improving temper stability. The nearly constant hardness of the 0.2 V steel over the 475–550 °C range is attributed to a balance between two competing processes: precipitation strengthening from the continued nucleation and growth of VC particles during tempering, which increases hardness, and recovery of the martensite/bainite lath structure, which reduces hardness. The net effect is a relatively stable hardness, indicating good tempering resistance. However, no distinct secondary-hardening peak was observed within the 475–550 °C range; the hardness remained approximately constant rather than showing a clear increase. We limit this observation to the temperature range investigated and avoid generalizing beyond 550 °C. In practical applications, P20 plastic mold steel typically requires a hardness maintained around 30 HRC. Based on tempering hardness data, 525 °C was selected as the uniform tempering temperature for this experiment, facilitating subsequent comparative analysis of microstructure and properties.

### 3.2. Microstructural Characterization and Comparison

#### 3.2.1. The Effect of Different Alloying Elements on the Tempering Microstructure of P20

**Figure 2:**
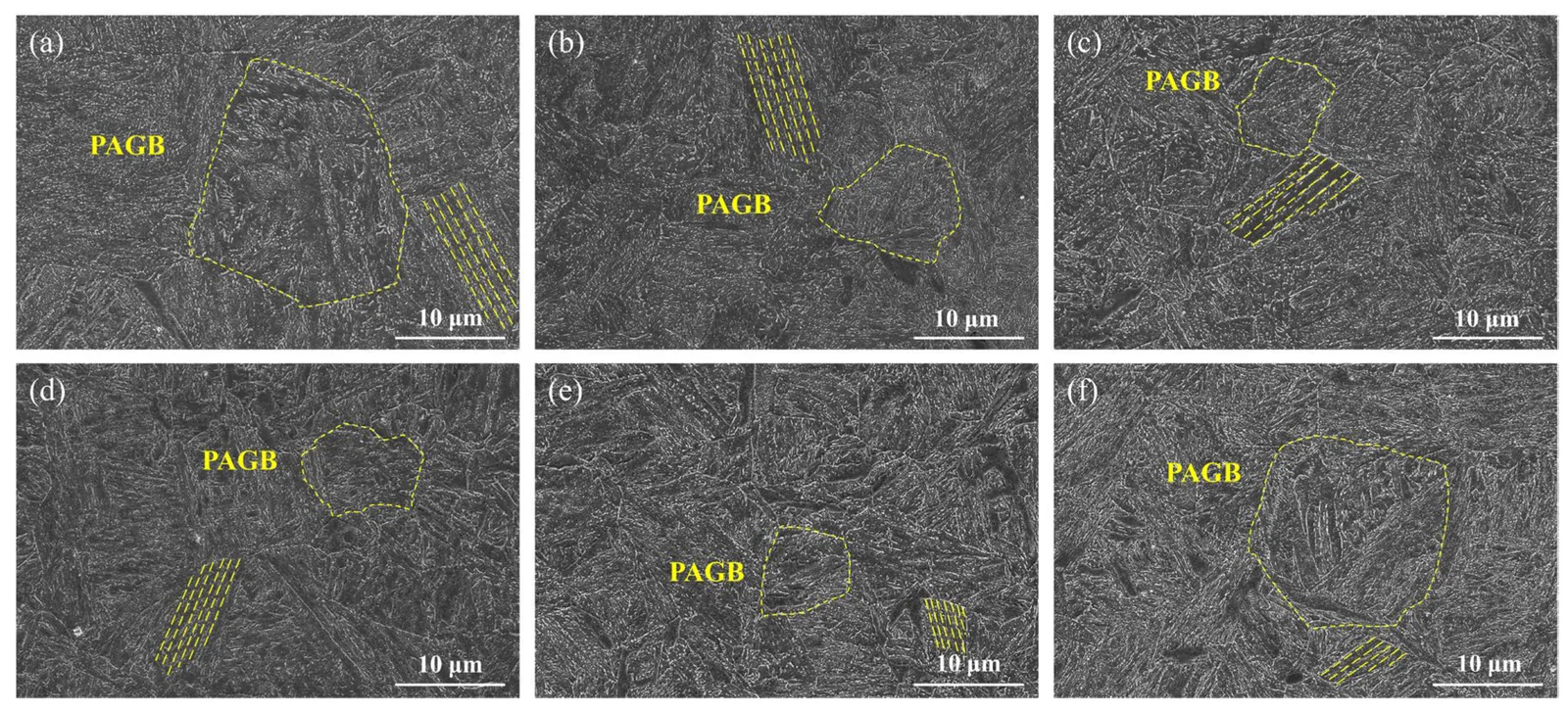
SEM images of different alloyed specimens (**a**) P20 original composition specimen; (**b**) 0.2 V specimen; (**c**) 0.1 Nb specimen; (**d**) 0.75 Si specimen; (**e**) 0.7 Mo specimen; (**f**) 1.5 Mn specimen.

**Figure 3:**
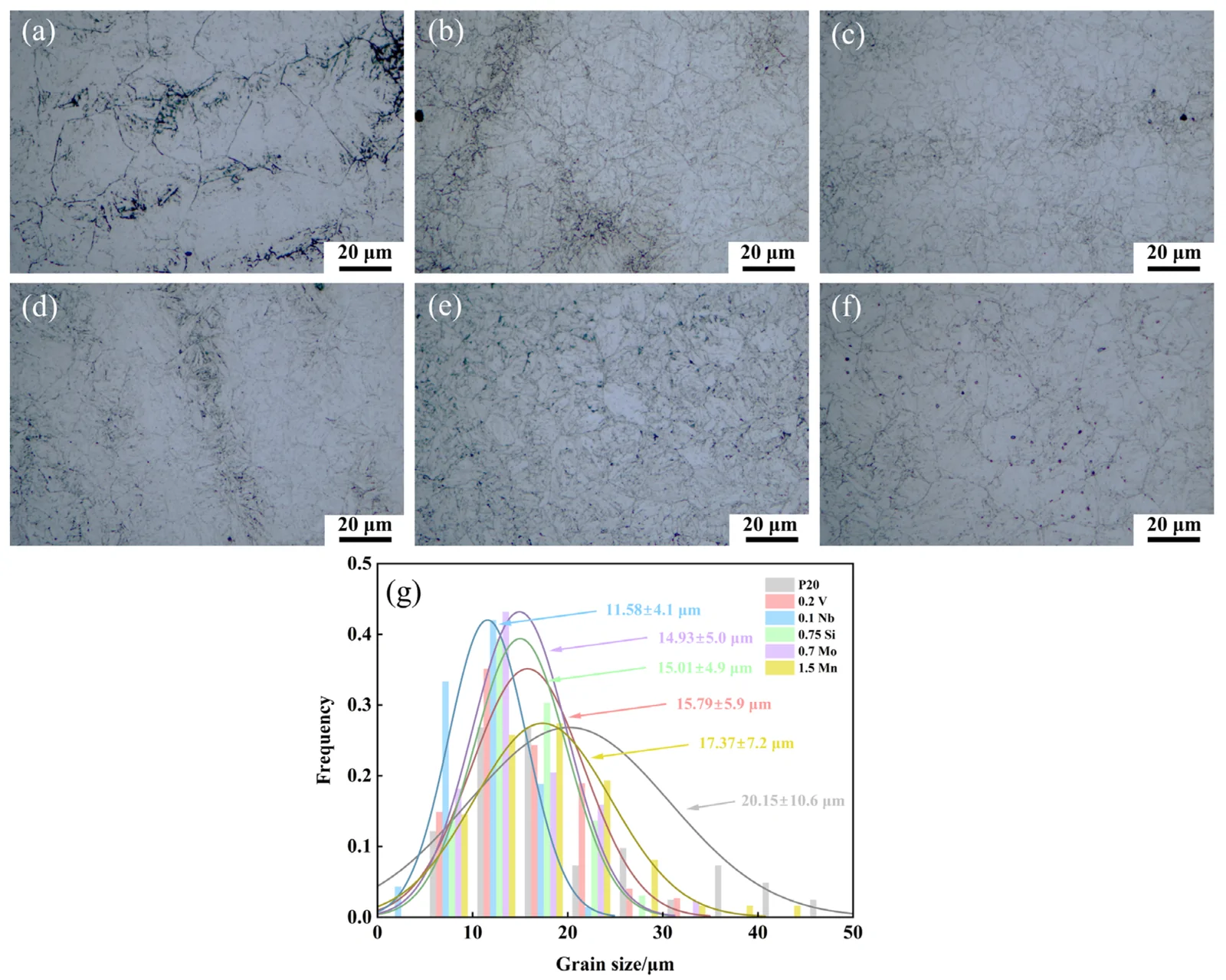
Grain size of different alloyed specimens (**a**) P20 original composition specimen; (**b**) 0.2 V specimen; (**c**) 0.1 Nb specimen; (**d**) 0.75 Si specimen; (**e**) 0.7 Mo specimen; (**f**) 1.5 Mn specimen; (**g**) grain size distribution curve.

Figure 2 shows the SEM microstructures of six different alloyed specimens after tempering at 525 °C. As shown in Figure 2, compared to the original P20 specimen, the prior austenite grain boundaries (PAGBs) of specimens with different alloying elements added all exhibit varying degrees of refinement. Among them, the 0.1 Nb and 0.7 Mo samples exhibited the most pronounced refinement of PAGB. The microstructure and size distribution statistics of the original austenite grains in each steel, obtained by etching with picric acid alcohol solution, are shown in Figure 3. The prior austenite grain sizes of the 0.1 Nb and 0.7 Mo samples decreased to 11.58 μm and 14.93 μm, respectively. The refinement effects of the 0.2 V and 0.75 Si samples are relatively similar, with average grain sizes of 15.01 μm and 15.79 μm, respectively. The Mn element exhibits the weakest refinement effect, with the prior austenite grain size of the 1.5 Mn sample being 17.37 μm, showing the smallest reduction. V and Nb, as strong carbide-forming elements, can effectively refine the prior austenite grain size by pinning grain boundaries through precipitate phases, thereby providing pronounced grain boundary strengthening. Solute elements such as Mo, Si, and Mn suppress grain boundary migration via solute drag effects, thus contributing to grain refinement. Studies have shown [24] that Mo can enhance austenite strength by refining austenite grain size, and the finer the austenite strength, the smaller the subsequent bainite lath dimensions. Previous studies [25] indicate that the microstructure of P20 mold steel after air cooling consists mainly of lamellar bainite and lath martensite. During tempering, carbides in the bainite tend to spheroidize and grow, while those in the martensite preferentially nucleate and precipitate at the lath boundaries. As a result, the steel’s microstructure after tempering still retains the characteristic lath morphology of the original air-cooled structure, as shown in the figure below.

**Figure 4:**
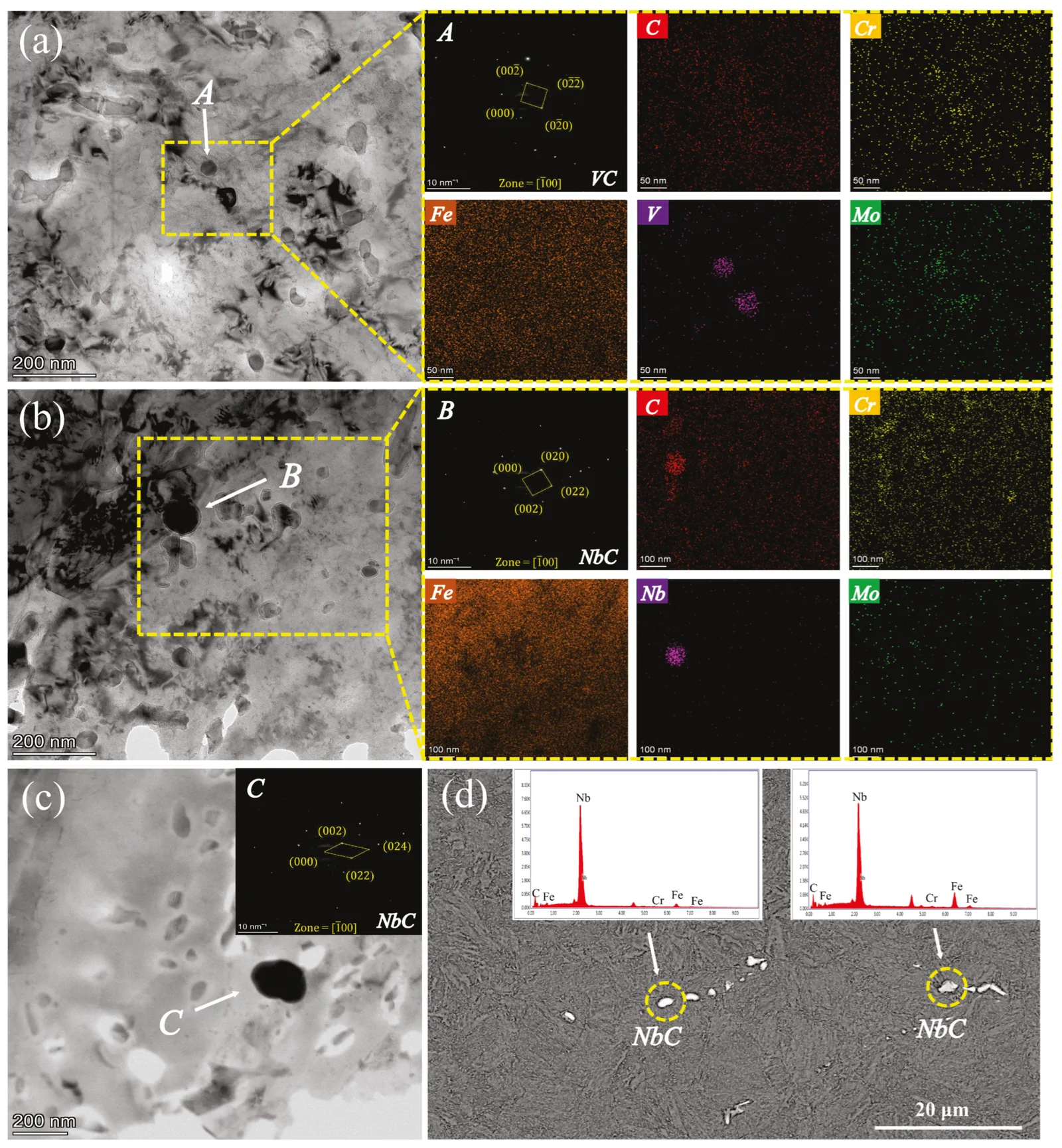
(**a**) TEM image of the 0.2 V sample; (**b**,**c**) TEM image of the 0.1 Nb sample; (**d**) SEM image of the 0.1 Nb sample.

**Figure 5:**
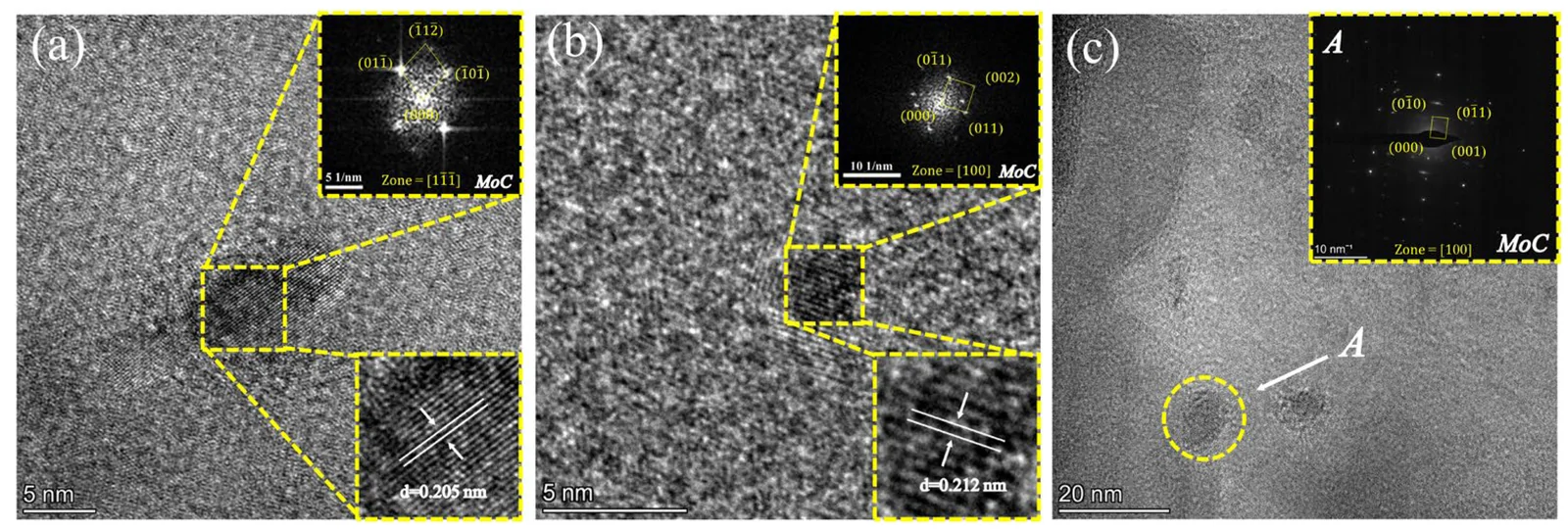
(**a**–**c**) TEM images of the 0.7 Mo sample.

The crystal structure of the precipitated carbides in the 0.2 V, 0.1 Nb, and 0.7 Mo samples was characterized using TEM and selected area electron diffraction (SAED) patterns, as shown in Figure 4. Fine precipitate particles with a size of approximately 50 nm were observed in the 0.2 V sample. SAED pattern indexing and energy-dispersive spectroscopy analysis confirmed that the precipitate phase is a (V-Mo)C-type carbide. In addition, no large-sized VC particles were observed in the examined TEM areas. Gong et al. [26] indicated that in steels containing both V and Mo, Mo can dissolve into various carbides and promote the formation of (V-Mo)C-type composite carbides. Additionally, the addition of Mo greatly refines the precipitate size and increases their number density and volume fraction. As shown in Figure 4b,c, precipitate particles with a size of approximately 100–200 nm are present in the 0.1 Nb sample, and SAED analysis confirms them to be NbC. Furthermore, as seen in the SEM image of Figure 4d, numerous elongated chain-like particles can also be observed in the 0.1 Nb sample; EDS analysis identifies these as micrometer-sized NbC particles. As shown in Figure 5, HRTEM and SAED analysis reveal that numerous extremely fine MoC particles, with diameters of approximately 2–10 nm, are distributed within the 0.7 Mo sample.

To further investigate the role of V and Nb in P20 plastic mold steel, the solubility formula was used to calculate the concentrations of dissolved V and Nb elements in austenite at an austenitizing temperature of 860 °C. The solubility formula is shown below [27]:

(1) $$\log\left(\left[V\right]\cdot \left[C\right]\right)=6.72-\frac{9500}{T}$$

(2) $$\log\left(\left[Nb\right]\cdot \left[C\right]\right)=2.96-\frac{7510}{T}$$

where $\left[V\right],\left[C\right],\left[Nb\right]$ are the mass fractions (wt.%) of solute V, C, and Nb in P20 plastic mold steel, respectively; T is temperature in K. Meanwhile, the V/C and Nb/C ratios in VC-type and NbC-type carbides are as follows:

(3) $$\frac{V_{VC}}{C_{VC}}=4.24$$

(4) $$\frac{{Nb}_{NbC}}{C_{NbC}}=7.74$$

Therefore, in 0.2 V steel and 0.1 Nb steel, the following equation holds:

(5) $$\frac{0.2-\left[V\right]}{0.32-\left[C\right]}=4.24$$

(6) $$\frac{0.1-\left[Nb\right]}{0.32-\left[C\right]}=7.74$$

By separately combining the above equation with the solubility formula, we obtain:

$$\left[V\right]=0.0748,\left[C\right]=0.2904$$

$$\left[Nb\right]=0.0007,\left[C\right]=0.3072$$

It should be noted that these solubility calculations are based on idealized assumptions and do not account for competitive precipitation with Cr- or Mo-rich carbides or local segregation. They are therefore presented as approximate thermodynamic estimates rather than as direct measurements of the actual precipitate fractions.

The above results indicate that in the 0.2 V sample, the mass fraction of V dissolved in austenite is 0.0748 wt.%, accounting for 37.4% of the total V content (0.2 V), Solubility estimation indicates that approximately 62.6% of V exists in the form of undissolved VC; TEM observations show that these particles are primarily less than 50 nm in size. In the 0.1 Nb sample, the mass fraction of Nb dissolved in austenite is 0.0007 wt.%, accounting for 0.7% of the total Nb content (0.1 Nb), solubility calculations indicate that approximately 99.3% of Nb exists in the form of undissolved NbC; TEM/SEM observations reveal that these particles exhibit a dual-scale distribution, with coarse ones ranging from 1–3 μm and fine ones from 100–200 nm.

As shown in Figure 4 and supported by thermodynamic calculations, the role of V in the 0.2 V sample manifests in two ways: first, most of the V exists as undissolved VC particles that pin austenite grain boundaries, resulting in marked grain refinement strengthening; second, approximately 0.0748 wt.% of dissolved V redisperses as nanoscale VC precipitates during tempering at 525 °C, providing strong precipitation strengthening. In the 0.1 Nb sample, the solubility product calculation indicates that 99.3% of Nb exists as undissolved NbC particles, exhibiting a dual-scale distribution ranging from micrometer to nanometer levels, which strongly pin grain boundaries and refine the microstructure. However, due to the low solubility of Nb at 860 °C, insufficient Nb solute is available during tempering for secondary precipitation, resulting in a lower contribution to precipitation hardening compared to V.

#### 3.2.2. The Analysis of EBSD

**Figure 6:**
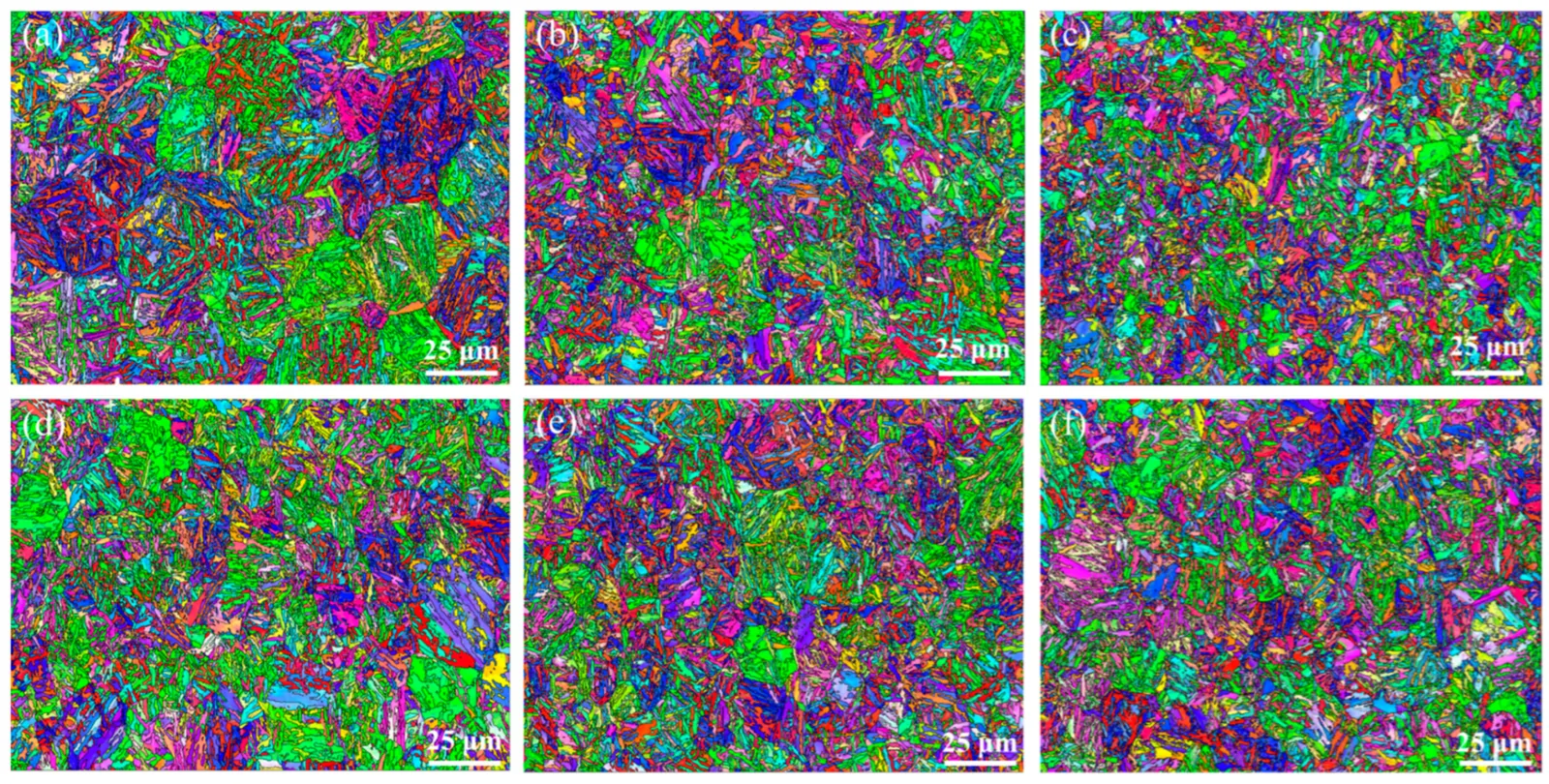
Inverse pole figures (IPFs) of different alloyed specimens (**a**) P20 specimen; (**b**) 0.2 V specimen; (**c**) 0.1 Nb specimen; (**d**) 0.75 Si specimen; (**e**) 0.7 Mo specimen; (**f**) 1.5 Mn specimen.

The inverse pole figures (IPFs) of six alloyed specimens tempered at 525 °C were plotted using EBSD, as shown in Figure 6. The original P20 specimen exhibited relatively large grain and lath sizes; however, with the addition of alloying elements, the grain and lath sizes of all specimens decreased to varying degrees. The effective grain size d of the six alloyed specimens was obtained by EBSD, and the data are shown in Table 2. With the addition of alloying elements, the effective grain size was refined to varying degrees. The samples with 0.1 Nb and 0.7 Mo exhibited an effective grain size of 2.43 μm, a reduction of approximately 9% compared to the original P20 composition sample’s 2.67 μm, showing the most marked refinement effect. The effective grain sizes of the 0.2 V, 0.75 Si, and 1.5 Mn specimens were 2.59 μm, 2.52 μm, and 2.49 μm, respectively, representing refinements of 3%, 5.6%, and 6.7% compared to the original P20 composition specimen. Refining the original austenite grain size can further reduce the martensite/bainite lath dimensions, thereby simultaneously refining the effective grain size. This is consistent with previous research findings [28,29].

**Figure 7:**
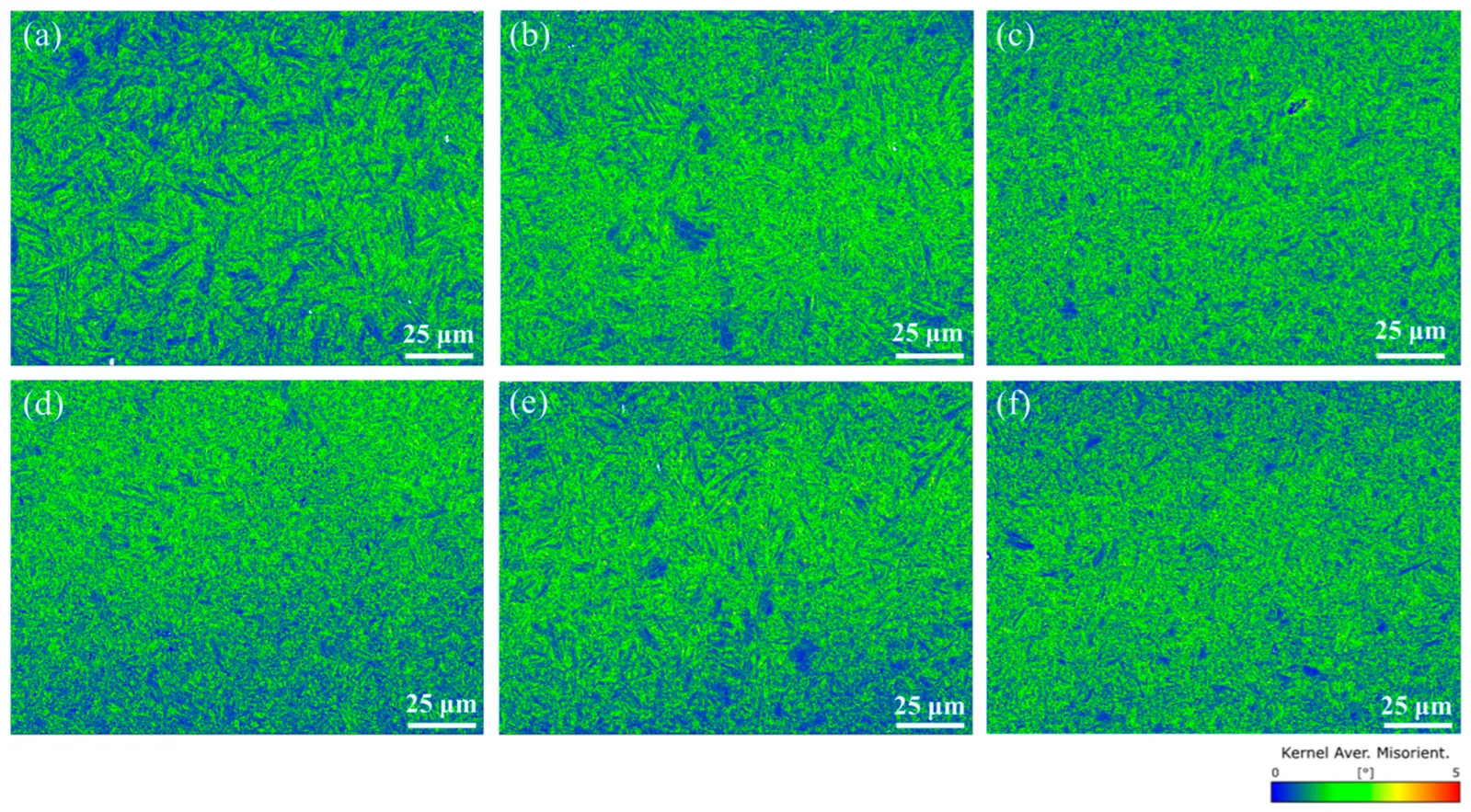
KAM maps of different alloyed specimens (**a**) P20 original composition sample; (**b**) 0.2 V sample; (**c**) 0.1 Nb sample; (**d**) 0.75 Si sample; (**e**) 0.7 Mo sample; (**f**) 1.5 Mn sample.

Figure 7 shows the KAM maps of six alloyed specimens. The KAM map is proportional to the geometrically necessary dislocation density (GND) and exhibits similar distribution characteristics, making it an effective tool for characterizing GND. The local orientation difference data (KAM) obtained from EBSD can be used to calculate the geometrically necessary dislocation density (GND), using the following formula [30]:

(7) $$\rho^{GND}=\frac{2\theta }{\mu b}$$

where: $\rho^{GND}$ is the geometrically necessary dislocation density within the region; θ is the average KAM value within the region; μ is the EBSD sampling step size; and b is the Burgers vector length of the material, with a value of 0.248 nm. Table 3 shows the geometrically necessary dislocation density calculated for the six alloyed samples.

It should be noted that KAM-derived GND density captures only the geometrically necessary dislocation component and excludes statistically stored dislocations; therefore, it represents a lower-bound estimate of the total dislocation density. Comparing the KAM maps with the geometrically necessary dislocation density table shows that, with the addition of alloying elements, the dislocation densities of the 0.2 V, 0.1 Nb, 0.75 Si, 0.7 Mo, and 1.5 Mn samples all increased to varying degrees, reaching 4.95 × 10^14^ m^−2^, 4.86 × 10^14^ m^−2^, 4.65 × 10^14^ m^−2^, 4.7 × 10^14^ m^−2^ and 4.7 × 10^14^ m^−2^, respectively. Among these, the 0.2 V sample showed the largest increase, while the 0.75 Si sample exhibited the smallest increase. V and Nb elements can hinder dislocation motion by precipitating fine carbides that pin dislocation lines. In contrast, previous studies have suggested that Si, Mo, and Mn atoms can segregate to dislocation lines and exert solute-drag effects that delay recovery [31,32]. The addition of these alloying elements effectively delays or suppresses the recovery process of the matrix during tempering, thereby indirectly increasing dislocation density.

### 3.3. Mechanical Property

#### 3.3.1. Comparison of Room Temperature Tensile Properties

**Figure 8:**
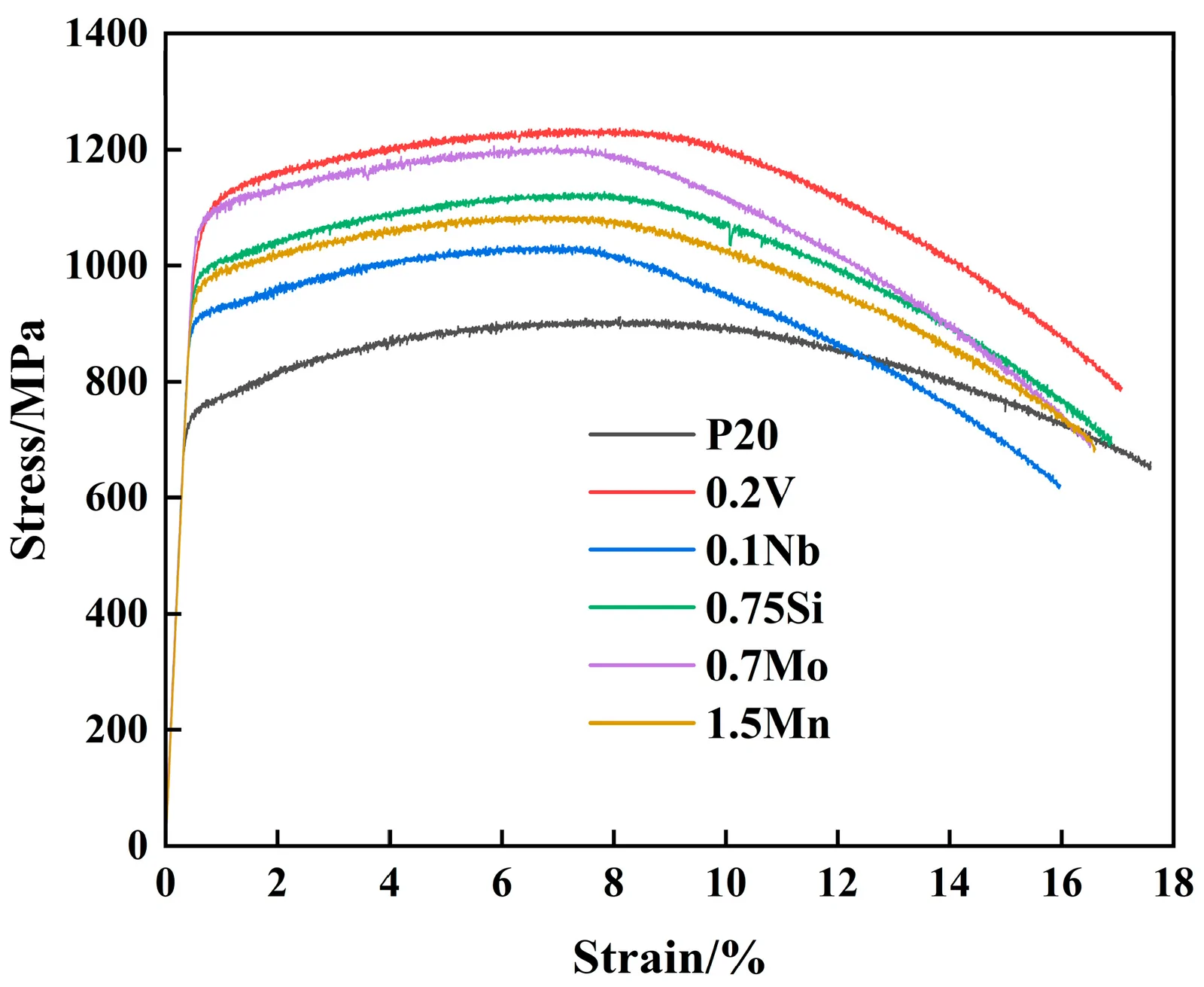
Stress–strain curves of different alloyed specimens.

Figure 8 shows the stress–strain curves of different alloyed specimens. All stress–strain curves show no distinct yield plateau, exhibiting characteristics of continuous yielding. Table 4 shows the variation trends in yield strength, tensile strength, elongation, reduction in area, and product of strength and elongation (PSE) for samples with different alloying compositions. Yield strength is defined as the 0.2% proof stress according to GB/T 228.1-2021. The stress–strain curves presented in Figure 8 are engineering stress–strain curves. Strain was measured using an extensometer attached to the gauge section. The product of strength and elongation (PSE) is defined as PSE (GPa·%) = (Tensile Strength, MPa × Elongation A_25_, %)/1000. This metric is commonly used to assess the strength–ductility balance in structural steels. The results indicate that the tensile strength of all alloyed samples was improved compared to the original P20 sample. Among them, the 0.2 V sample exhibited the highest tensile strength of 1237 MPa, representing a 35.7% increase over the original P20 sample’s 911 MPa. Next was the 0.7 Mo sample, which reached a tensile strength of 1207 MPa, an increase of 32.5% compared to the original P20 sample. The 0.75 Si sample achieved a tensile strength of 1127 MPa, a 23.7% improvement over the baseline. The tensile strengths of the 1.5 Mn and 0.1 Nb samples were 1088 MPa and 1036 MPa, respectively, corresponding to increases of 19.4% and 13.7% compared to the P20 sample. In terms of ductility, the P20 original composition specimen exhibited the highest elongation (A_25_), reaching 17.64%. The remaining specimens ranked in descending order of elongation as follows: 0.2 V specimen (17.04%), 1.5 Mn specimen (16.52%), 0.75 Si specimen (16.40%), 0.1 Nb specimen (16.08%), and 0.7 Mo specimen (16.04%). The trend in specific product of strength and elongation (PSE) was generally consistent with that of tensile strength, with the 0.2 V specimen achieving the highest value of 21.07 GPa·%, followed by the 0.7 Mo specimen at 19.36 GPa·%, representing increases of 31.1% and 20.5%, respectively, compared to the P20 specimen. Based on these results, the 0.2 V and 0.7 Mo specimens demonstrated superior combinations of strength and ductility.

#### 3.3.2. Fracture Surface Morphology Analysis

**Figure 9:**
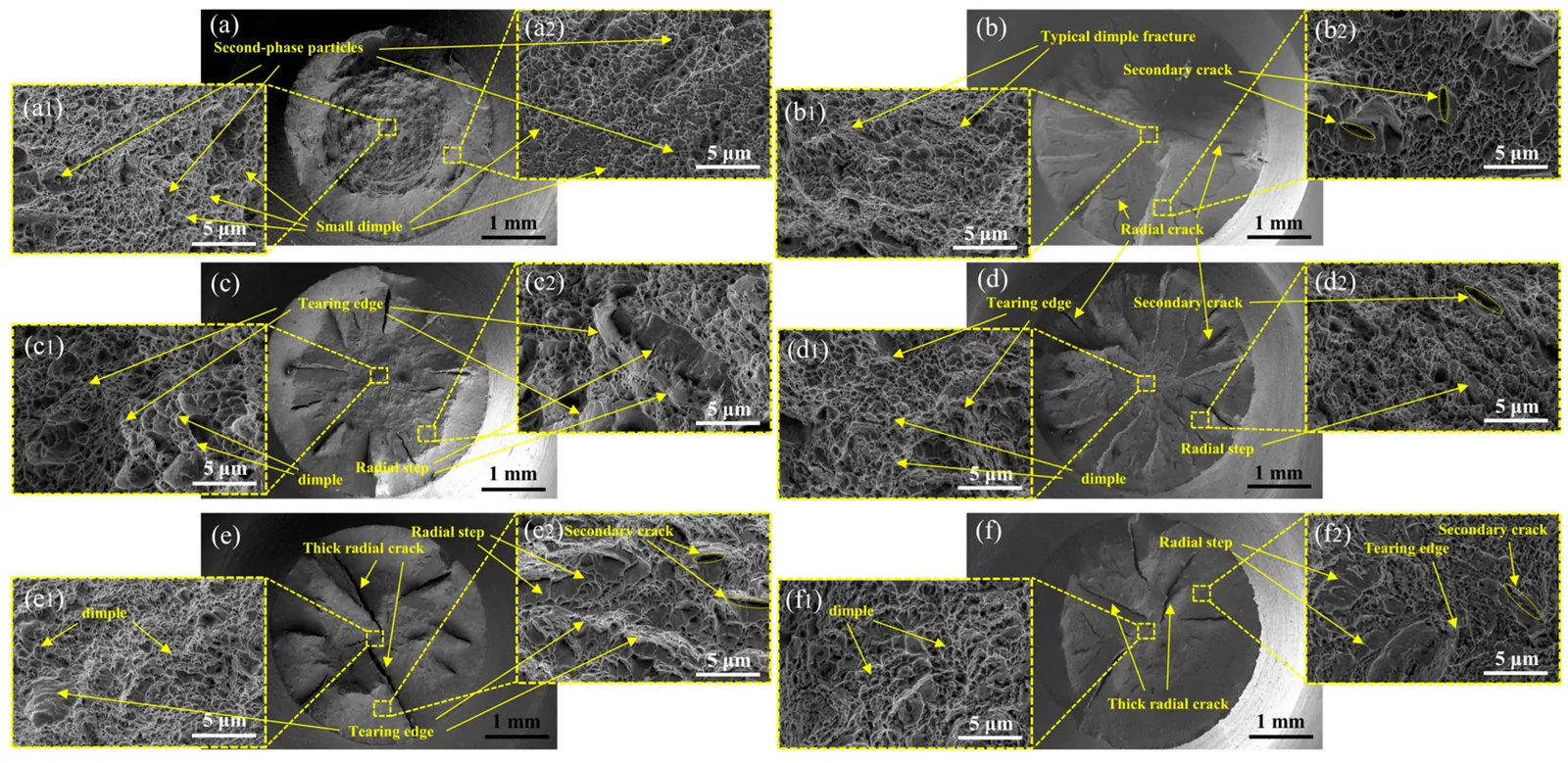
Fracture surface morphology of different alloyed specimens (**a**–**f**) macroscopic fracture surfaces; (**a1**–**f1**) crack initiation zone morphology; (**a2**–**f2**) crack propagation zone morphology.

Macroscopic and microscopic fracture surface observations were conducted on tensile specimens of six different alloying compositions, as shown in Figure 9, to reveal their fracture mechanisms. The macroscopic fracture surfaces of all specimens exhibited typical ductile fracture morphology, consisting of three regions: a central fibrous zone (crack initiation zone), a radiating ring zone (crack propagation zone), and an outermost shear lip (final fracture zone). The plastic behavior of different specimens shows pronounced differences, as analyzed by combining macroscopic fracture characteristics with elongation (A_25_). The crack propagation zone of the P20 original composition specimen showed no obvious radial cracks, and had the widest shear lip, indicating optimal ductility, with an A_25_ value of 17.64%. The 0.2 V specimen exhibited fine radial cracks in the propagation zone, with a slightly reduced shear lip width, resulting in a minor decrease in ductility, with an A_25_ of 17.04%. The 0.1 Nb and 0.75 Si specimens displayed distinct radial cracks in their crack propagation zones, with crack lengths reaching up to 1 mm or even longer, further reducing ductility, with A_25_ values of 16.08% and 16.40%, respectively. The 0.7 Mo specimen had numerous cracks in the propagation zone, including a few coarse cracks approximately 2 mm long, leading to slightly lower ductility, with an A_25_ of 16.04%. The 1.5 Mn specimen showed crack lengths in the propagation zone similar to those of the 0.75 Si specimen (approximately 1 mm), with comparable ductility, achieving an A_25_ of 16.52%. The observed fracture surface features are generally consistent with the measured elongation values. However, because the elongation differences are small and not all pairwise comparisons are statistically significant, we do not attribute specific fracture morphology differences directly to the A_25_ ranking. The fractographic analysis is presented primarily as a qualitative description of fracture mechanisms.

The central fiber region of the P20 original composition specimen consists of numerous fine and uniform dimples (Figure 9(a1)); in the radial zone, dimple size slightly increases, with a few large dimples formed by second-phase particles observed, but no distinct cleavage steps are evident overall (Figure 9(a2)), exhibiting typical ductile fracture characteristics with good plasticity. The 0.2 V specimen still predominantly exhibits ductile dimple fracture, but the dimple density in the fiber region is lower than that of P20, and the dimple size has slightly increased (Figure 9(b1)). A small number of secondary cracks appear in the crack propagation zone (Figure 9(b2)), yet a large number of dimples remain, indicating reduced plasticity compared to the P20 specimen. In the fiber region of the 0.1 Nb specimen, continuous tearing ridges and polygonal dimples were observed (Figure 9(c1)); the crack propagation zone exhibited a river-like fracture surface morphology (Figure 9(c2)), with numerous cleavage steps, indicating quasi-cleavage fracture characteristics and further reduced ductility. The micro-fracture morphology of the 0.75 Si and 1.5 Mn samples is relatively similar. Both exhibit distinct continuous tearing ridges and numerous fine dimples in the fibrous zone (Figure 9(d1,f1)); cleavage steps and secondary cracks are observed in the crack propagation zone (Figure 9(d2,f2)). Compared to the P20 sample, both show reduced ductile fracture characteristics and enhanced brittle fracture features, with similar plasticity. Although fine dimples are still visible in the fiber region of the 0.7 Mo specimen, the tear ridges are coarse (Figure 9(e1)); the crack propagation zone exhibits distinct river-like patterns, numerous secondary cracks, and cleavage steps (Figure 9(e2)), showing the most prominent quasi-cleavage fracture characteristics, resulting in reduced elongation.

With the addition and adjustment of alloying elements, the fracture surface morphology of the specimens gradually transitions from typical ductile fracture characterized by microvoid coalescence to quasi-cleavage fracture. Among them, the specimen with the original P20 composition exhibits the best ductility; the 0.2 V specimen shows slightly reduced ductility but still maintains ductile fracture behavior; the specimens with 0.1 Nb, 0.75 Si, and 1.5 Mn exhibit characteristics of quasi-cleavage fracture, with further decreased ductility; the 0.7 Mo specimen displays the strongest tendency toward brittleness, yet based on tensile test data, it still retains relatively good ductility.

#### 3.3.3. Impact Performance and Impact Fracture Surface Analysis

**Figure 10:**
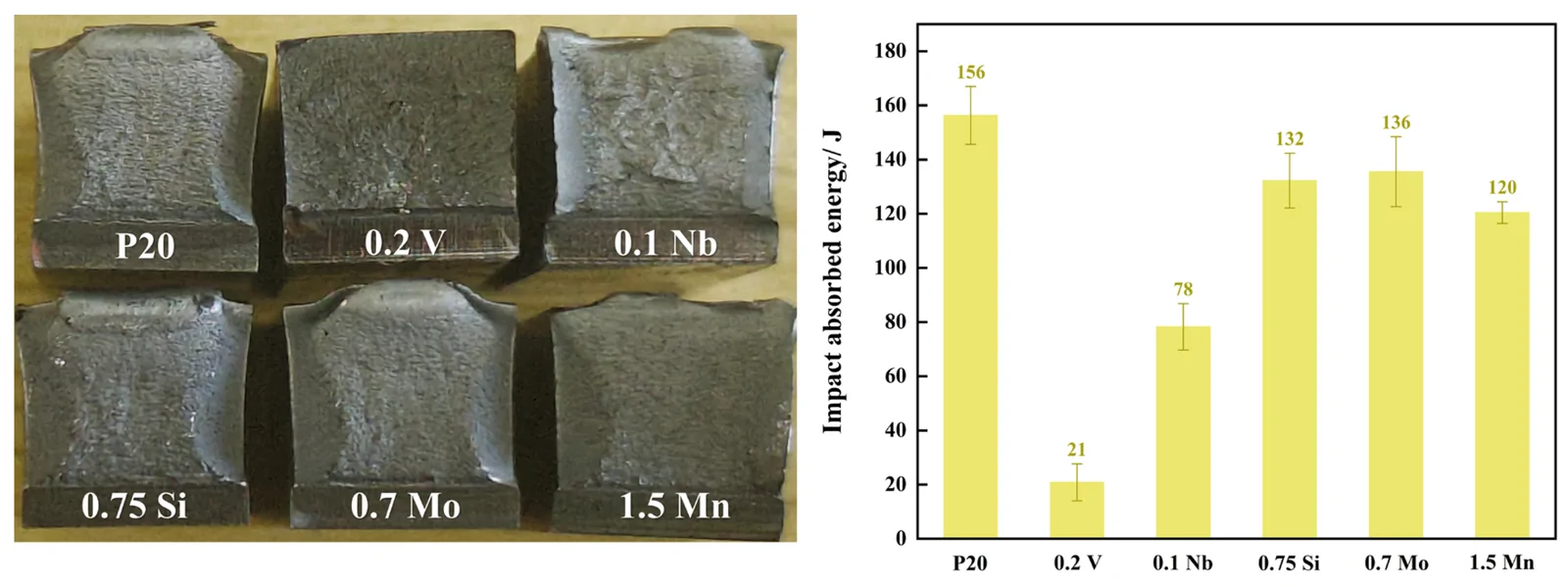
Macroscopic impact fracture morphology and impact energy of different alloyed specimens.

Figure 10 shows the macroscopic impact fracture morphology and impact properties of six alloyed specimens. The impact fracture surface mainly consists of three regions: a central fibrous zone originating from crack initiation at the notch bottom, a rapidly propagating radial zone, and a final shear lip region where fracture occurs. As shown in the figure, in the P20, 0.75 Si, 0.7 Mo, and 1.5 Mn specimens, the fibrous zone and shear lip region occupied the majority of the fracture surface area, exhibiting clear ductile fracture characteristics, with impact energies of 156 J, 132 J, 136 J, and 120 J, respectively. In the 0.2 V specimen, the fibrous zone was almost invisible, showing distinct brittle fracture features, with the impact energy greatly reduced to 21 J. The 0.1 Nb specimen exhibited a fibrous zone and shear lip area fraction between the above two cases, and its impact energy also fell between them at 78 J, indicating that this specimen is undergoing a transition from ductile to brittle fracture.

**Figure 11:**
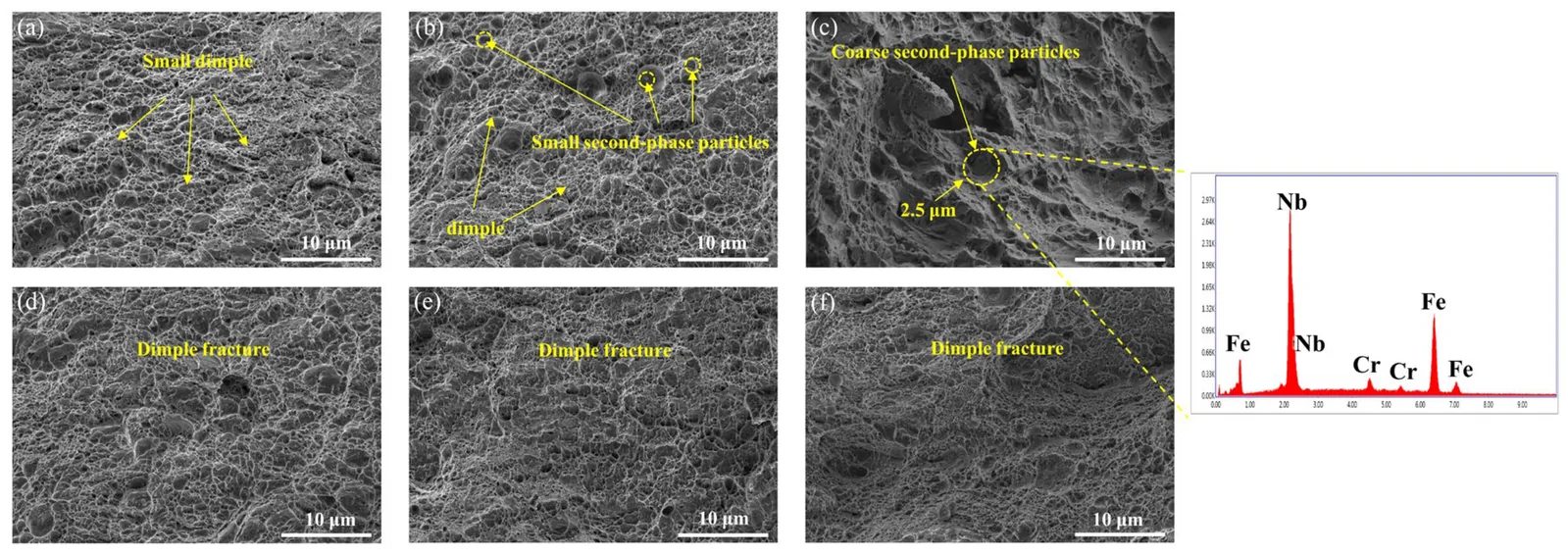
Morphology of the fibrous zone in impact fracture surfaces of different alloyed specimens (**a**) P20 original composition specimen; (**b**) 0.2 V specimen; (**c**) 0.1 Nb specimen; (**d**) 0.75 Si specimen; (**e**) 0.7 Mo specimen; (**f**) 1.5 Mn specimen.

Figure 11 shows the micrographs of the fibrous zone in the impact fracture surfaces of six different alloying compositions. As shown in Figure 11a,d–f, the central fibrous zones of the P20, 0.75 Si, 0.7 Mo, and 1.5 Mn specimens consist of numerous fine dimples, exhibiting typical ductile fracture characteristics. The dimple size of the 0.2 V specimen is larger compared to that of the P20 specimen, and numerous microvoids induced by fine secondary-phase particles are present within (Figure 11b). However, due to the extremely small size of these secondary-phase particles, composition characterization via energy-dispersive spectroscopy (EDS) was not feasible. Nevertheless, considering the addition of 0.2 V in this specimen and relevant literature reports [33], these fine particles inferred to be V-rich MC-type carbides. In contrast, the 0.1 Nb specimen contains relatively large secondary-phase particles, as shown in Figure 11c, with spherical particles approximately 2.5 μm in diameter. EDS analysis indicates that these particles are coarse NbC.

**Figure 12:**
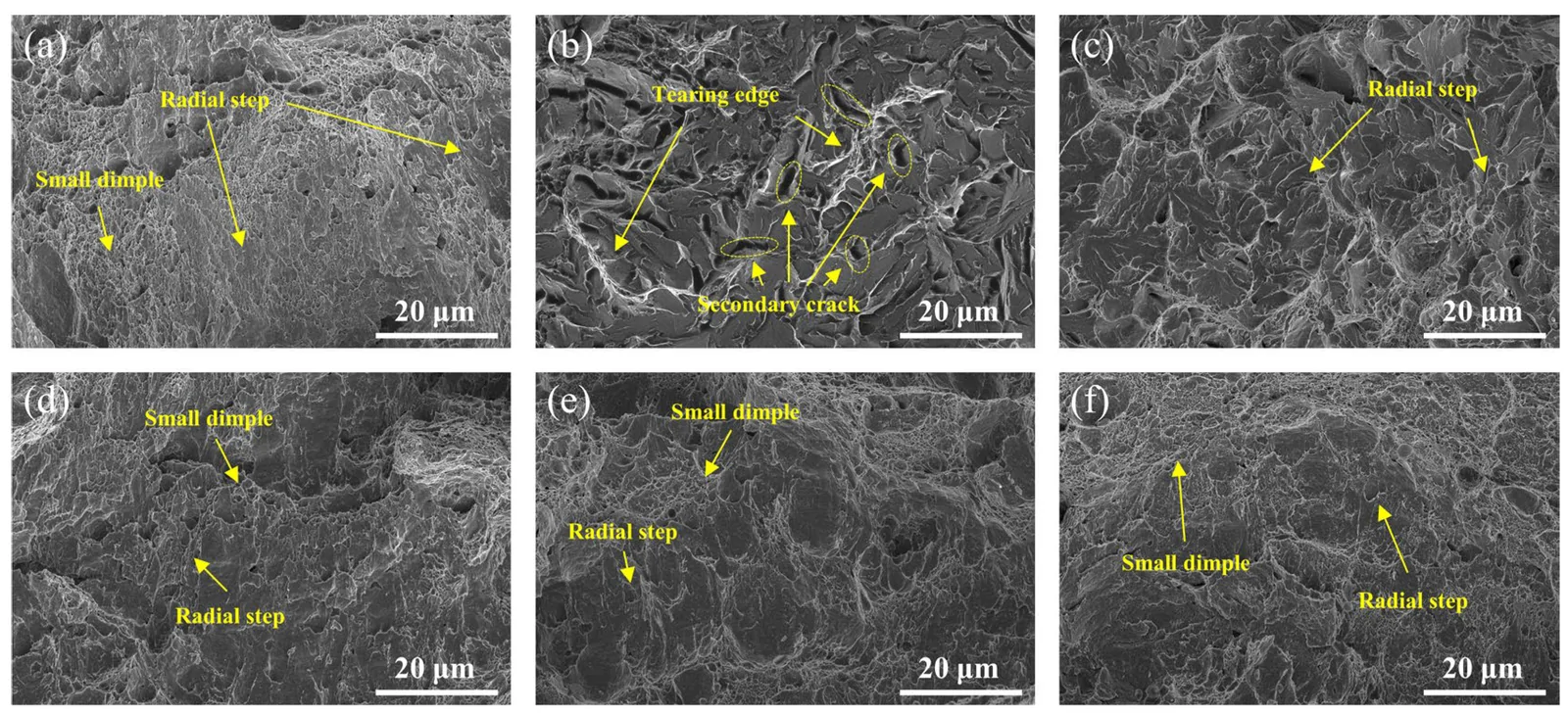
Morphology of the radiating zone in impact fracture surfaces of different alloyed specimens (**a**) P20 original composition specimen; (**b**) 0.2 V specimen; (**c**) 0.1 Nb specimen; (**d**) 0.75 Si specimen; (**e**) 0.7 Mo specimen; (**f**) 1.5 Mn specimen.

Figure 12 shows the micrographs of the radiating zone on the impact fracture surfaces of six different alloying compositions. In the P20, 0.75 Si, 0.7 Mo, and 1.5 Mn samples, a large number of dimples were still observed in the fracture region, accompanied by cleavage steps and river-like cleavage patterns, indicating that all four samples exhibit good toughness. The 0.2 V sample showed distinct large-scale tearing ridges, along whose edges numerous fine tearing patterns extended, and a large number of secondary cracks were also observed, exhibiting typical brittle fracture characteristics. The tearing ridge size in the 0.1 Nb sample was smaller compared to that in the 0.2 V sample, yet numerous cleavage steps remained visible, suggesting improved toughness relative to the 0.2 V sample, although the improvement was limited, and the fracture mechanism belonged to quasi-cleavage fracture.

**Figure 13:**
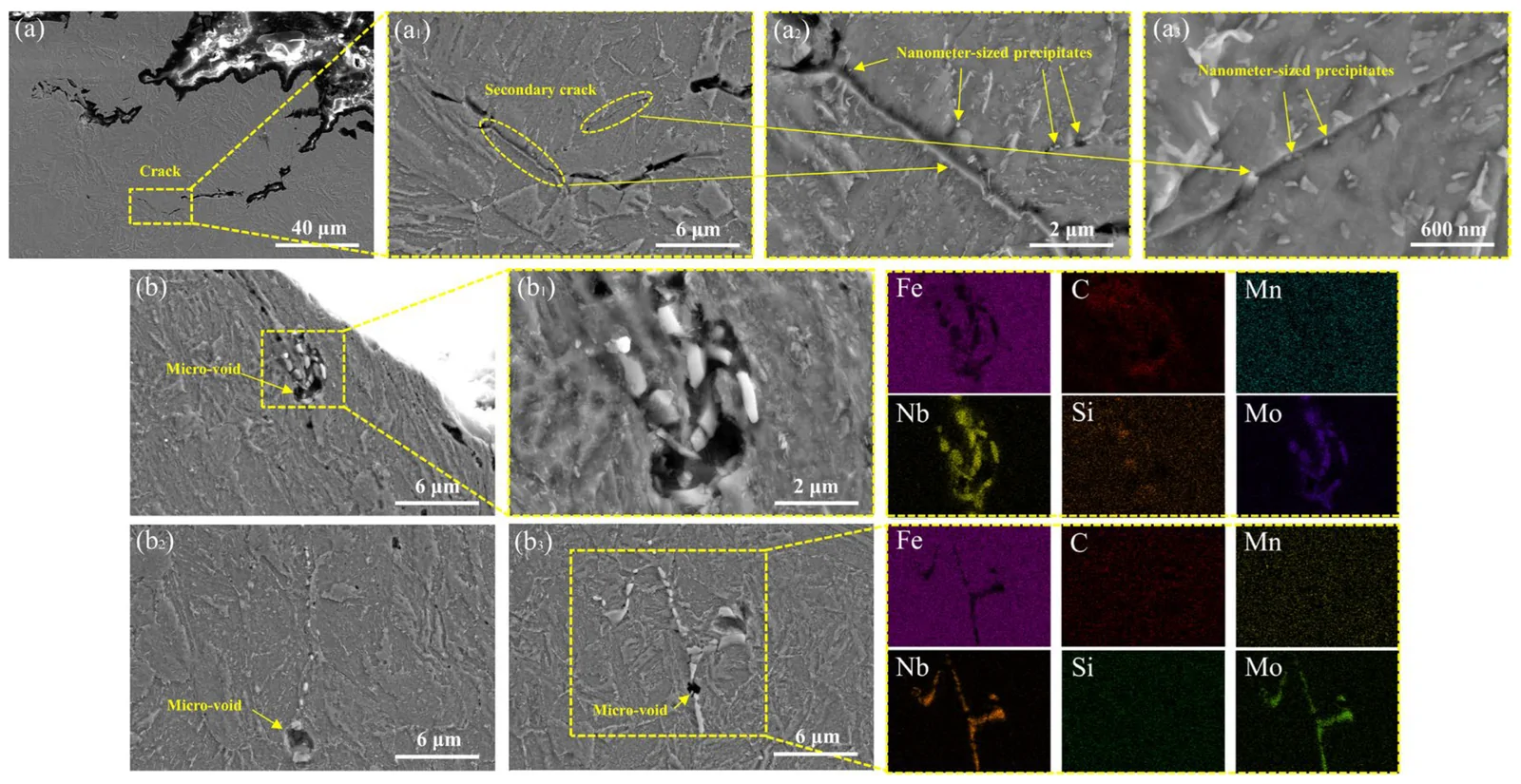
Longitudinal impact fracture surface morphology of different alloyed specimens (**a**,**a1**–**a3**) 0.2 V specimen; (**b**,**b1**–**b3**) 0.1 Nb specimen.

To further investigate the impact crack propagation behavior of six different alloyed specimens, the fracture surfaces were longitudinally sectioned for microstructural observation. Figure 13 shows the longitudinal cross-sectional morphology of the impact fracture surfaces for the 0.2 V and 0.1 Nb samples. As shown in Figure 13a, large-scale cracks are present in the cross-section of the 0.2 V sample, and fine secondary cracks have originated from the main crack during its propagation. Further observation of Figure 13(a1–a3) reveals a large number of fine spherical nanoscale precipitates within the secondary cracks. Meanwhile, the dislocation density ($\rho^{GND})$ of the 0.2 V sample is the highest among the six alloyed samples, reaching 4.95 × 10^14^ m^−2^. Generally, microstructure refinement—including the reduction in both original austenite grain size and effective grain size—helps improve material toughness and lower the ductile-to-brittle transition temperature. However, in the 0.2 V sample, the impact energy decreased by more than 80%, and the fracture mode shifted from ductile to brittle fracture. Previous studies [15] have shown that although vanadium (V) contributes to grain refinement, excessive addition leads to non-uniform distribution of V in the matrix and promotes the formation of eutectic carbides VC, thereby deteriorating impact properties. In this experiment, a large number of dispersed nanoscale VC particles greatly enhanced strength by pinning dislocations; however, the resulting high density of immobile dislocations tends to cause stress concentration and dislocation pile-ups in localized regions, leading to the generation of numerous secondary cracks during fracture and severely degrading the material’s impact toughness. Moreover, the pronounced increase in strength is also a major reason for the sharp decline in toughness.

Generally, the impact fracture process begins with the nucleation of micro-void, which primarily form around second-phase particles (such as carbides). As shown in Figure 13(b,b1–b3), numerous micro-voids exist in the cross-section of the 0.1 Nb sample. Figure 13(b1) reveals that a large number of coarse second-phase particles are aggregated within these micro-voids. EDS analysis indicates that these particles consist of NbC phase. Furthermore, as illustrated in Figure 13(b2,b3), elongated chain-like distributions of second-phase particles can also initiate micro-void formation, further increasing the risk of brittle fracture. EDS analysis similarly confirms that these chain-like particles belong to either NbC or Nb-Mo composite precipitate phases. During the austenitization process of the 0.1 Nb specimen, coarse Nb-rich particles remained undissolved after austenitizing and ultimately remain in the microstructure at room temperature. During fracture, cracks tend to propagate along these coarse NbC particles, leading to brittle fracture and a pronounced reduction in impact energy.

**Figure 14:**
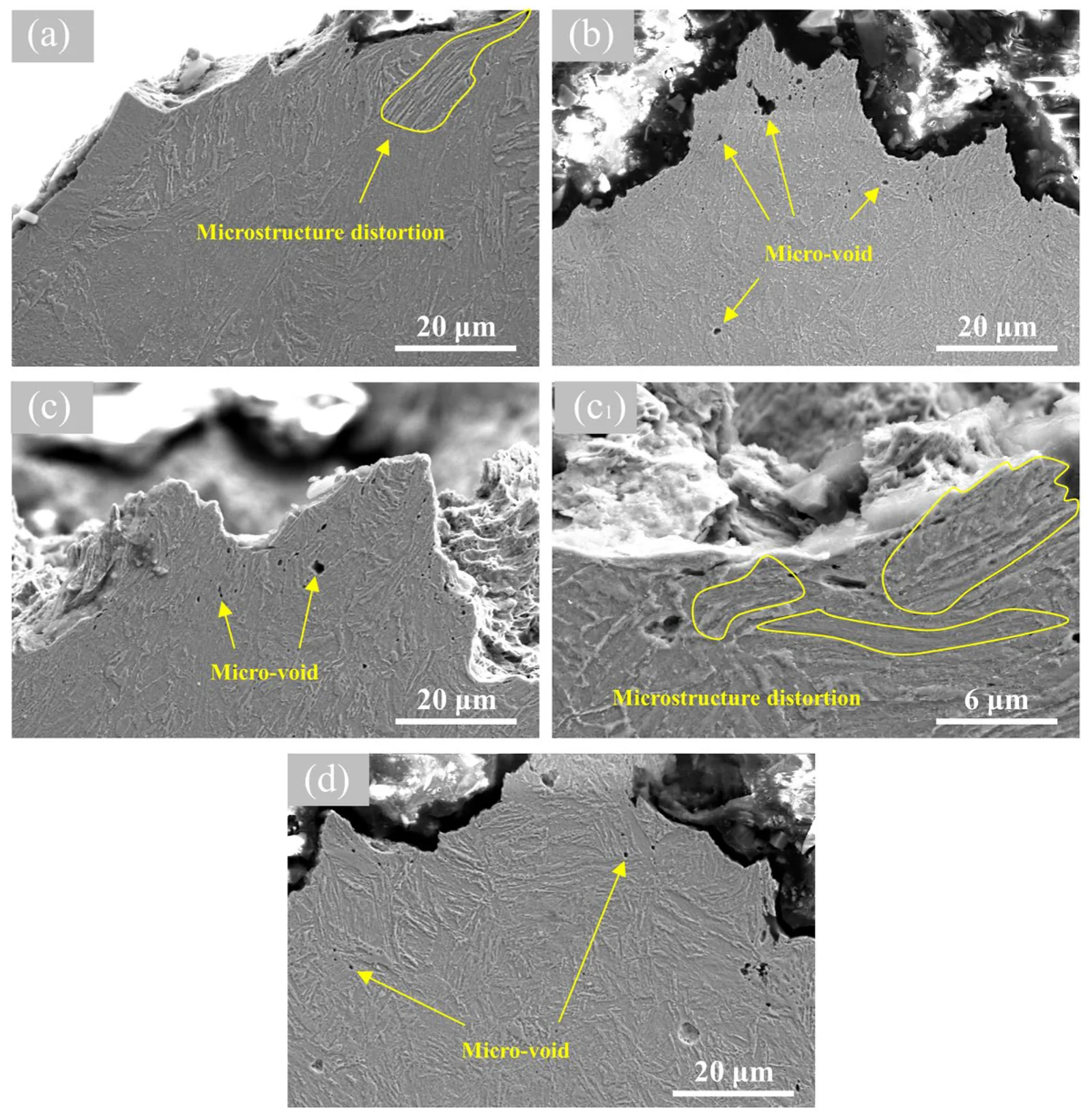
Longitudinal impact fracture surface morphology of different alloyed specimens (**a**) P20 original composition specimen; (**b**) 0.75 Si specimen; (**c**,**c1**) 0.7 Mo specimen; (**d**) 1.5 Mn specimen.

Figure 14 shows the longitudinal cross-sectional morphology of the impact fracture surface for the P20, 0.75 Si, 0.7 Mo, 1.5 Mn specimen. As shown in Figure 14a, the fracture surface of the P20 original composition specimen is clean, with no obvious micro-void observed in the examined fields, consistent with its high impact toughness. In contrast, specimens with 0.75 Si (Figure 14b), 0.7 Mo (Figure 14c), and 1.5 Mn (Figure 14d) exhibit a small number of micro-voids on their surfaces, but no distinct secondary cracks are present, and no coarse second-phase particles are found within the micro-voids, suggesting that all three still maintain relatively good toughness levels. Further observation of the P20 (Figure 14a) and the 0.7 Mo specimen (Figure 14(c1)) reveals microstructural distortion near the fracture surface, with the lath-like structure showing bending, indicating that the matrix possesses good ductility and toughness.

Furthermore, the tensile elongation of the 0.7 Mo steel (16.04%) is only marginally lower than the other specimens (16.08–17.64%), and these differences are within experimental scatter. Regarding the fracture morphology, the tensile fractographs of the 0.7 Mo steel show some quasi-cleavage features; however, this is a typical macroscopic fracture appearance for high-strength tempered martensitic/bainitic steels under tensile loading and does not necessarily indicate poor intrinsic toughness. Thus, the observed difference between tensile and impact fracture behavior reflects different loading conditions and fracture mechanisms involved.

### 3.4. Strengthening Mechanism

For the six steel grades in this experiment, their high yield strength results from a combination of multiple strengthening mechanisms. The total yield strength is contributed by solid solution strengthening, grain boundary strengthening, dislocation strengthening, and precipitation strengthening, as described by the following formula [34,35,36]:

(8) $$\sigma_{YS}=∆\sigma_{0}+∆\sigma_{ss}+∆\sigma_{GB}+\sqrt{\left({{∆\sigma }_{DIS}}^{2}+{{∆\sigma }_{Prec}}^{2}\right)}$$

where: $\sigma_{YS}$ is the yield strength (MPa); $∆\sigma_{0}$ is the intrinsic strength of the matrix (MPa), approximately 88 MPa [37,38]; $∆\sigma_{ss}$ is the solid solution strengthening contribution (MPa); $∆\sigma_{GB}$ is the grain boundary strengthening contribution (MPa); ${∆\sigma }_{DIS}$ is the dislocation strengthening contribution (MPa); and ${∆\sigma }_{Prec}$ is the precipitation strengthening contribution (MPa). Among these, substitutional and interstitial alloying elements can produce solid solution strengthening effects, and the increment due to solid solution strengthening can be obtained from the following equation [39]:

(9) $$∆\sigma_{ss}=32.34\left[Mn\right]+83.16\left[Si\right]+360.3\left[C\right]+33\left[Ni\right]+11\left[Mo\right]+354.2\left[N\right]$$

where $\left[Mn\right],\;\left[Si\right],\;\left[C\right],\;\left[Ni\right],\left[Mo\right],[N]$ represent the mass fractions (wt.%) of the solute elements, respectively. Since the solubility of C in tempered martensite/bainite is low, its contribution to solid solution strengthening can be approximately considered as zero. The solid solution strengthening contributions for each steel grade calculated using the above formula are shown in Table 5.

The grain boundary strengthening amount $∆\sigma_{GB}$ can be calculated using the Hall-Petch equation [40]:

(10) $$∆\sigma_{GB}=K_{HP}\cdot d^{-\frac{1}{2}}$$

where $K_{HP}$ is 0.19 ${MPa\cdot m}^{1/2}$, and d is the effective grain size. The Hall–Petch coefficient $K_{HP}=$ ${0.19\;MPa\cdot m}^{1/2}$ was adopted from the literature on 718-type plastic mold steels [38], which share the same class of tempered martensite/bainite microstructures as the P20 steel investigated in this study. Lucía et al. [41] demonstrated that using a 15° misorientation angle as the criterion for grain boundaries, the measured bainite packet size can effectively suppress crack propagation. Wang et al. [42] demonstrated that the martensite lath width and the size of martensite block boundaries jointly determine the contribution of grain boundary strengthening, and used EBSD to mark the positions of martensite blocks, defining grain boundaries with an orientation difference greater than 15° as martensite block boundaries. Tian et al. [43] found that the effective grain size calculated from EBSD-measured grain boundaries with orientation differences exceeding 15° per unit area closely matches the block size, thus better reflecting the actual block size in steel. Therefore, d in the above equation represents the effective grain size measured within grain boundaries with misorientation angles greater than 15°, and the calculated grain boundary strengthening values are shown in Table 6:

The dislocation strengthening increment ${∆\sigma }_{DIS}$ is calculated using the following formula [38,44]:

(11) $${∆\sigma }_{DIS}=MG\alpha b\rho^{\frac{1}{2}}$$

where M is the Taylor factor with a value of 3; G is the shear modulus with a value of 78.5 GPa; α is a constant with a value of 0.25; b is the Burgers vector length with a value of 0.248 nm; ρ is the dislocation density of the sample, as shown in Table 3; substituting these values yields the dislocation strengthening contribution ${∆\sigma }_{DIS}$, as presented in Table 7:

The precipitation strengthening effect ${∆\sigma }_{Prec}$ is related to the volume fraction of precipitates and their interaction with dislocations, and can be calculated using the Orowan formula [45]:

(12) $${∆\sigma }_{Prec}=\frac{0.538Gbf^{\frac{1}{2}}}{X}\ln\frac{X}{2b}$$

(13) f=V·ρ

where: f is the volume fraction of the precipitate phase; X is the equivalent spherical diameter of the precipitate phase; b is the Burgers vector length, with a value of 0.248 nm; V is the volume of the precipitate phase; and ρ is the number density of the precipitate phase per unit volume. Since current studies mostly rely on TEM images to measure and calculate the precipitation strengthening contribution ${∆\sigma }_{Prec}$, the resulting values of ${∆\sigma }_{Prec}$ are subject to errors due to factors such as thin foil thickness and uneven distribution of precipitates in TEM samples. Therefore, this study determines the precipitation strengthening ${∆\sigma }_{Prec}$ by subtracting the intrinsic matrix strength $∆\sigma_{0}$, solid solution strengthening $∆\sigma_{ss}$, grain boundary strengthening $∆\sigma_{GB}$, and dislocation strengthening ${∆\sigma }_{DIS}$ from the total yield strength $\sigma_{YS}$, as shown in Table 8. It should be emphasized that the residual term assigned to ‘precipitation strengthening’ in this analysis is an estimate obtained by subtracting the other strengthening contributions from the experimental yield strength. Without independent quantitative characterization of precipitate size distributions, number densities, and volume fractions, this residual cannot be independently validated as precipitation strengthening. It may also include contributions from unmeasured solid-solution effects, retained austenite, lath/packet morphology, statistically stored dislocations, inclusions, and model errors. We therefore present this term as a model-dependent estimate rather than a directly measured precipitation strengthening contribution. In addition, all calculated strengthening values are approximate estimates. Given the uncertainties in input parameters and empirical coefficients, the reported values typically have a certain margin of error.

Because the residual term is determined by subtracting the estimated strengthening contributions from the experimental yield strength, the model is calibrated to match each experimental value and therefore lacks independent predictive validation. The model is intended as a comparative framework for assessing the relative contributions of different strengthening mechanisms across the six compositions, rather than as an independently validated predictive tool for absolute yield strength.

**Figure 15:**
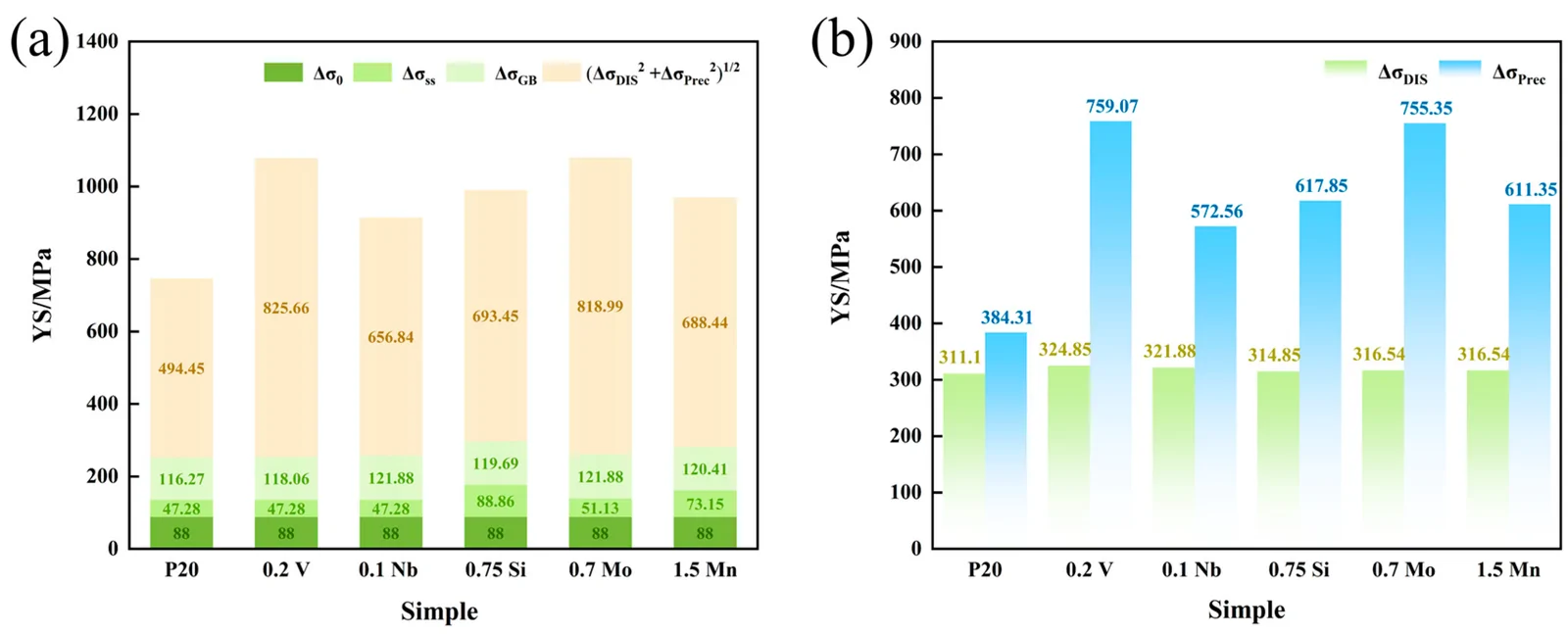
(**a**) Strengthening contributions of different alloyed samples; (**b**) Calculated contributions of dislocation strengthening and precipitation strengthening.

The combined contribution of six alloying elements to strength is shown in Figure 15, with dislocation strengthening and precipitation strengthening being the primary strengthening mechanisms for P20 plastic mold steel. The precipitation strengthening contribution of the 0.2 V sample is approximately twice that of the original P20 composition sample. The large number of finely dispersed VC particles effectively pin grain boundaries, impede dislocation motion, and refine the microstructure, resulting in good strength-ductility balance. However, due to a high density of immobile dislocations causing dislocation pile-ups, secondary crack propagation is triggered, greatly reducing the toughness. The contribution of precipitation strengthening in the 0.1 Nb sample was only slightly higher than that of the P20 and 0.75 Si samples. The dual-scale distribution (nanoscale and microscale) of NbC consumed a large amount of carbon, while the microscale carbides had limited effect on enhancing steel strength, resulting in relatively low strength and poor toughness of the material.

As a ferrite-strengthening element, Si enhances the strength of steel through strong solid solution strengthening. In addition, increasing the Si content helps refine the grain size by suppressing the growth of ferrite in bainite and refining the lath microstructure, thereby achieving grain refinement strengthening [46]. The 0.75 Si specimen achieved effective strengthening of P20 plastic mold steel through the synergistic effects of multiple strengthening mechanisms, resulting in a favorable balance between strength and ductility. Mn is a strong austenite-stabilizing element that increases the activation energy for self-diffusion of carbon and iron atoms, thereby reducing their diffusion coefficients and greatly suppressing diffusion-controlled pearlite transformation, thus improving hardenability. Additionally, studies have shown [47] that increasing Mn content can substantially reduce bainitic lath size and refine the microstructure, which is consistent with the results of this experiment. Therefore, the 1.5 Mn specimen also exhibits good combinations of strength and ductility.

Increasing the Mo content enhances the solute drag effect, hinders grain boundary migration, and refines the grain size. As shown in Table 2, the effective grain size of the 0.7 Mo specimen is the same as that of the 0.1 Nb specimen, indicating that 0.7 Mo and 0.1 Nb have similar effects on microstructure refinement. During tempering, a high Mo content enables the precipitation of a large amount of finely dispersed MoC particles, which are extremely fine and effectively hinder dislocation movement, resulting in strong precipitation strengthening. In summary, the 0.7 Mo specimen exhibits the best overall performance.

## 4. Conclusions

In this study, the effects of five alloying elements (V, Nb, Si, Mo, and Mn) on the microstructural evolution and mechanical properties of P20 plastic mold steel have been systematically investigated, and the underlying strengthening and toughening mechanisms have been identified. The main conclusions are drawn as follows:

(1) Among the six compositions tested, the 0.2 V steel exhibited the highest tensile strength (1237 MPa) but the lowest impact toughness (21 J). The severe toughness loss is correlated with high dislocation density and stress concentration around fine V-Mo-rich carbides promoting secondary cracking.
(2) The 0.7 Mo steel achieved the best overall strength–toughness balance, with a tensile strength of 1207 MPa and impact energy of 136 J, due to the formation of abundant nanoscale Mo-rich carbides (2–10 nm) that provide effective precipitation strengthening without causing severe embrittlement.
(3) The 0.1 Nb steel showed only moderate strength improvement (1036 MPa) and limited impact toughness (78 J). The coarse undissolved NbC particles (1–3 μm) and chain-like Nb-rich precipitates acted as crack initiation sites, restricting both strength and toughness enhancement.
(4) The 0.75 Si and 1.5 Mn steels exhibited intermediate tensile strengths of 1127 MPa and 1088 MPa, respectively, with impact energies of 132 J and 120 J. Their strengthening arose primarily from solid-solution and grain refinement effects, with more moderate impacts on toughness compared to V and Nb additions.
(5) This comparative study demonstrates that 0.7 Mo steel provides the best overall strength–toughness balance, while 0.2 V steel offers the highest strength but at the cost of severely degraded toughness. These findings provide experimental guidance for the compositional design of high-performance prehardened plastic mold steels.

## Figures and Tables

**Table 1:** Nominal and measured chemical composition of the test steel (wt.%).

Steel		C	Mn	Cr	Mo	Si	Al	V	Nb
P20	Nominal	0.32	0.7	1.80	0.35	0.25	0.025	-	-
	Measured	0.32	0.73	1.83	0.35	0.27	0.023	-	-
0.2 V	Nominal	0.32	0.7	1.80	0.35	0.25	0.025	0.20	-
	Measured	0.33	0.72	1.72	0.35	0.27	0.025	0.19	-
0.1 Nb	Nominal	0.32	0.7	1.80	0.35	0.25	0.025	-	0.1
	Measured	0.32	0.73	1.73	0.35	0.27	0.025	-	0.1
0.75 Si	Nominal	0.32	0.7	1.80	0.35	0.75	0.025	-	-
	Measured	0.33	0.72	1.70	0.35	0.74	0.025	-	-
0.7 Mo	Nominal	0.32	0.7	1.80	0.70	0.25	0.025	-	-
	Measured	0.32	0.72	1.69	0.68	0.25	0.025	-	-
1.5 Mn	Nominal	0.32	1.5	1.80	0.35	0.25	0.025	-	-
	Measured	0.33	1.46	1.73	0.35	0.26	0.025	-	-

**Table 2:** Effective grain size of different alloyed specimens.

	P20	0.2 V	0.1 Nb	0.75 Si	0.7 Mo	1.5 Mn
d/um	2.67	2.59	2.43	2.52	2.43	2.49

**Table 3:** Geometrically necessary dislocation density of different alloyed specimens.

	P20	0.2 V	0.1 Nb	0.75 Si	0.7 Mo	1.5 Mn
$\rho^{GND}$/m^−2^	4.54 × 10^14^	4.95 × 10^14^	4.86 × 10^14^	4.65 × 10^14^	4.7 × 10^14^	4.7 × 10^14^

**Table 4:** Tensile properties of different alloyed specimens.

Sample	Yield Strength (MPa)	Tensile Strength (MPa)	Elongation/A_25_ (%)	Reduction in Area (%)	PSE (GPa·%)
P20	746	911	17.64	61.68	16.07
0.2 V	1079	1237	17.04	60.41	21.07
0.1 Nb	914	1036	16.08	59.98	16.66
0.75 Si	990	1127	16.40	60.59	18.48
0.7 Mo	1080	1207	16.04	60.80	19.36
1.5 Mn	970	1088	16.52	60.81	17.97

**Table 5:** Contribution of Solid Solution Strengthening.

	P20	0.2 V	0.1 Nb	0.75 Si	0.7 Mo	1.5 Mn
$∆\sigma_{ss}$/MPa	47.28	47.28	47.28	88.86	51.13	73.15

**Table 6:** Contribution of Grain Boundary Strengthening.

	P20	0.2 V	0.1 Nb	0.75 Si	0.7 Mo	1.5 Mn
$∆\sigma_{GB}$/MPa	116.27	118.06	121.88	119.69	121.88	120.41

**Table 7:** Contribution of Dislocation Strengthening.

	P20	0.2 V	0.1 Nb	0.75 Si	0.7 Mo	1.5 Mn
${∆\sigma }_{DIS}$/MPa	311.1	324.85	321.88	314.85	316.54	316.54

**Table 8:** Contribution of Precipitation Hardening.

	P20	0.2 V	0.1 Nb	0.75 Si	0.7 Mo	1.5 Mn
${∆\sigma }_{Prec}$/MPa	384.31	759.07	572.56	617.85	755.35	611.35

## Data Availability

The original contributions presented in this study are included in the article. Further inquiries can be directed to the corresponding author.
